# Supplementary material for: Efficacy of Liver-Directed Combined Radiotherapy in Locally Advanced Hepatocellular Carcinoma with Portal Vein Tumor Thrombosis
Source: Cancers (Basel). 2023 Jun 13;15(12):3164. doi: 10.3390/cancers15123164 (PMC10296128; doi:10.3390/cancers15123164)
Supplement: Supplementary file 1 [file cancers-15-03164-s001.zip › cancers-2452888-supplementary/Supplementary Files/Supplementary figures.pptx]

## Slide 1
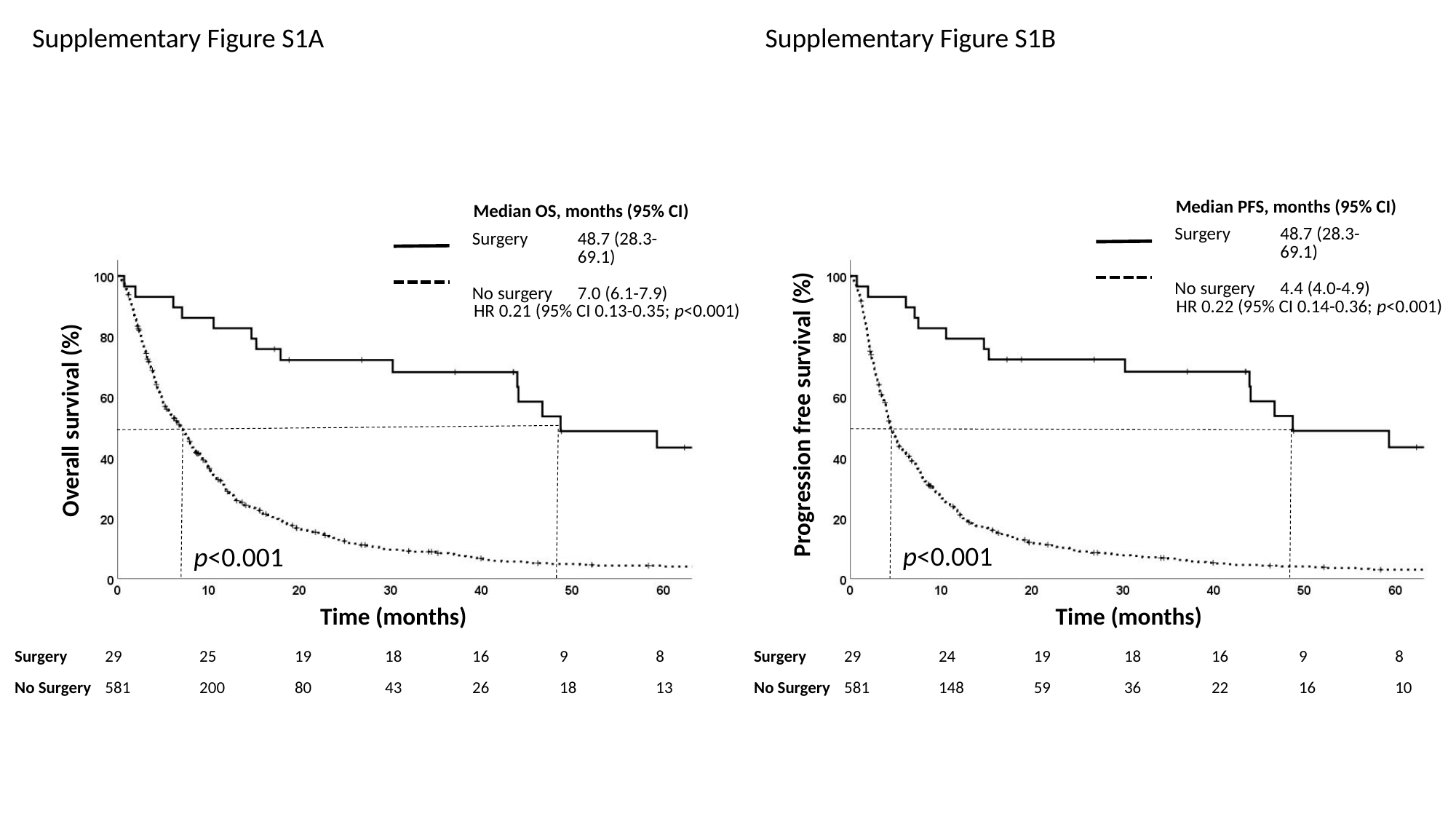

Supplementary Figure S1A
Supplementary Figure S1B
Median PFS, months (95% CI)
Median OS, months (95% CI)
| Surgery | 48.7 (28.3-69.1) |
| --- | --- |
| No surgery | 4.4 (4.0-4.9) |
| Surgery | 48.7 (28.3-69.1) |
| --- | --- |
| No surgery | 7.0 (6.1-7.9) |
Progression free survival (%)
HR 0.22 (95% CI 0.14-0.36; p<0.001)
HR 0.21 (95% CI 0.13-0.35; p<0.001)
Overall survival (%)
p<0.001
p<0.001
Time (months)
Time (months)
| Surgery | 29 | 24 | 19 | 18 | 16 | 9 | 8 |
| --- | --- | --- | --- | --- | --- | --- | --- |
| No Surgery | 581 | 148 | 59 | 36 | 22 | 16 | 10 |
| Surgery | 29 | 25 | 19 | 18 | 16 | 9 | 8 |
| --- | --- | --- | --- | --- | --- | --- | --- |
| No Surgery | 581 | 200 | 80 | 43 | 26 | 18 | 13 |

## Slide 2
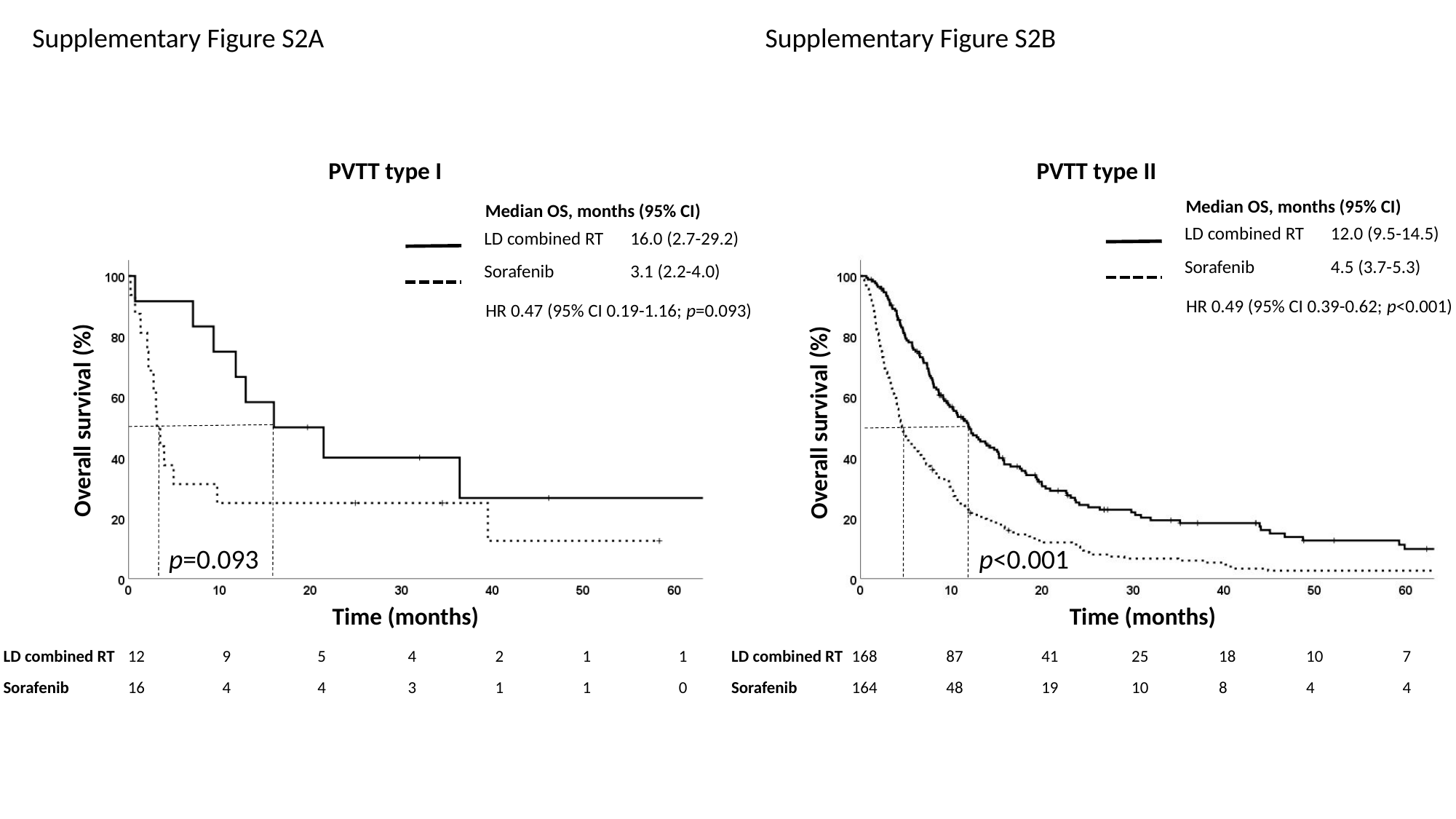

Supplementary Figure S2A
Supplementary Figure S2B
PVTT type I
PVTT type II
Median OS, months (95% CI)
Median OS, months (95% CI)
| LD combined RT | 12.0 (9.5-14.5) |
| --- | --- |
| Sorafenib | 4.5 (3.7-5.3) |
| LD combined RT | 16.0 (2.7-29.2) |
| --- | --- |
| Sorafenib | 3.1 (2.2-4.0) |
HR 0.49 (95% CI 0.39-0.62; p<0.001)
HR 0.47 (95% CI 0.19-1.16; p=0.093)
Overall survival (%)
Overall survival (%)
p=0.093
p<0.001
Time (months)
Time (months)
| LD combined RT | 12 | 9 | 5 | 4 | 2 | 1 | 1 |
| --- | --- | --- | --- | --- | --- | --- | --- |
| Sorafenib | 16 | 4 | 4 | 3 | 1 | 1 | 0 |
| LD combined RT | 168 | 87 | 41 | 25 | 18 | 10 | 7 |
| --- | --- | --- | --- | --- | --- | --- | --- |
| Sorafenib | 164 | 48 | 19 | 10 | 8 | 4 | 4 |

## Slide 3
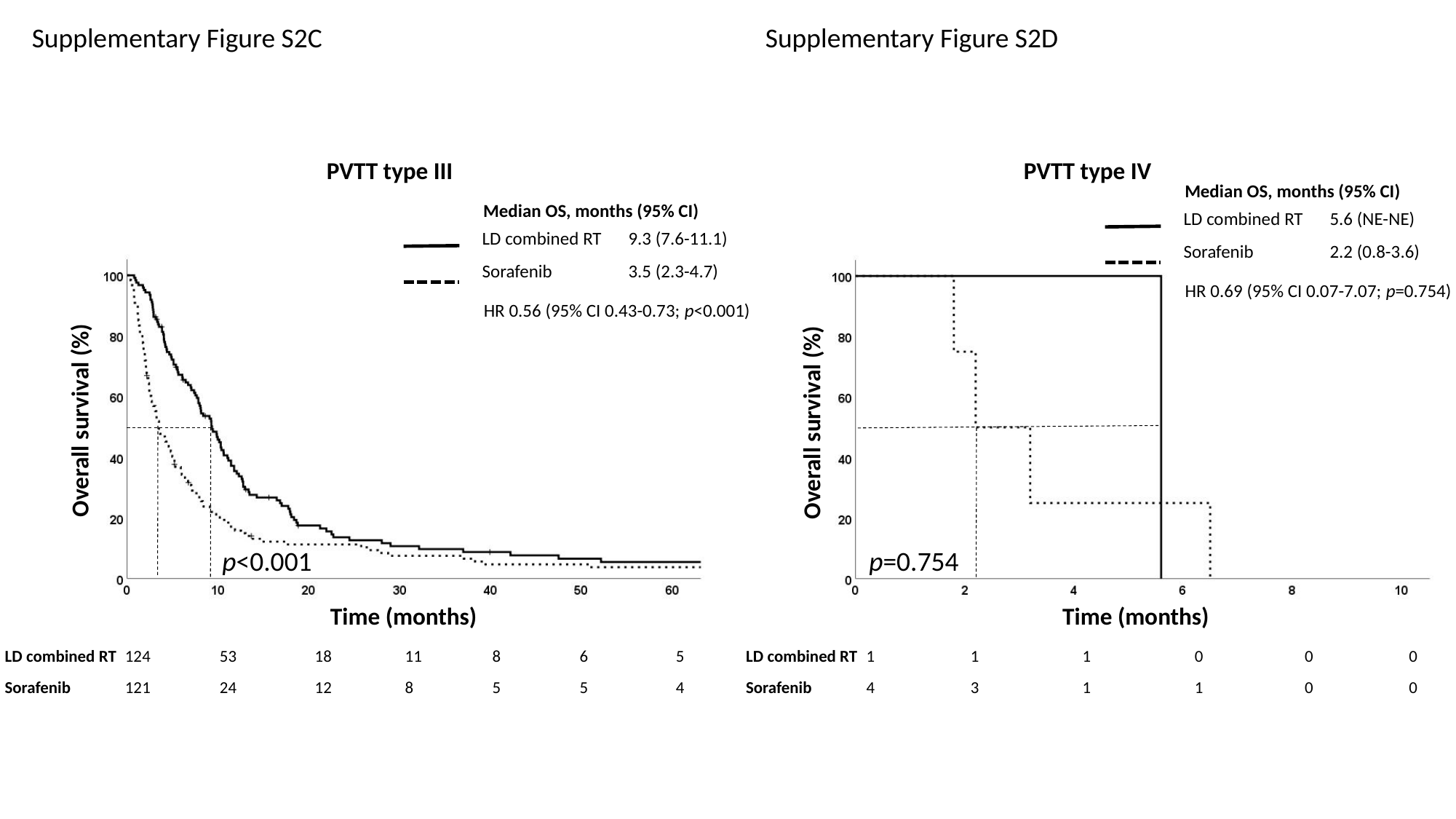

Supplementary Figure S2C
Supplementary Figure S2D
PVTT type III
PVTT type IV
Median OS, months (95% CI)
Median OS, months (95% CI)
| LD combined RT | 5.6 (NE-NE) |
| --- | --- |
| Sorafenib | 2.2 (0.8-3.6) |
| LD combined RT | 9.3 (7.6-11.1) |
| --- | --- |
| Sorafenib | 3.5 (2.3-4.7) |
HR 0.69 (95% CI 0.07-7.07; p=0.754)
HR 0.56 (95% CI 0.43-0.73; p<0.001)
Overall survival (%)
Overall survival (%)
p<0.001
p=0.754
Time (months)
Time (months)
| LD combined RT | 1 | 1 | 1 | 0 | 0 | 0 |
| --- | --- | --- | --- | --- | --- | --- |
| Sorafenib | 4 | 3 | 1 | 1 | 0 | 0 |
| LD combined RT | 124 | 53 | 18 | 11 | 8 | 6 | 5 |
| --- | --- | --- | --- | --- | --- | --- | --- |
| Sorafenib | 121 | 24 | 12 | 8 | 5 | 5 | 4 |

## Slide 4
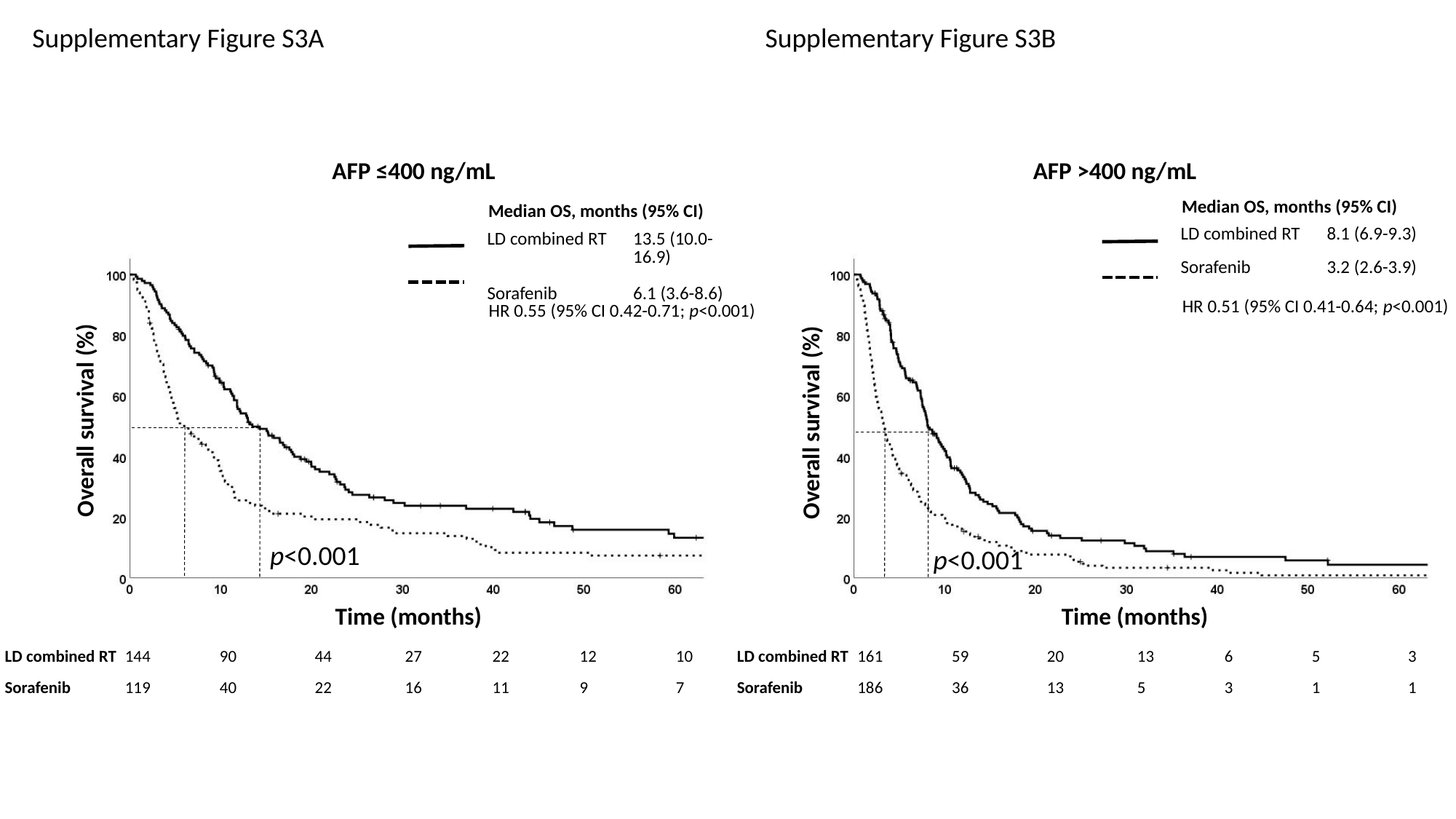

Supplementary Figure S3A
Supplementary Figure S3B
AFP ≤400 ng/mL
AFP >400 ng/mL
Median OS, months (95% CI)
Median OS, months (95% CI)
| LD combined RT | 8.1 (6.9-9.3) |
| --- | --- |
| Sorafenib | 3.2 (2.6-3.9) |
| LD combined RT | 13.5 (10.0-16.9) |
| --- | --- |
| Sorafenib | 6.1 (3.6-8.6) |
HR 0.51 (95% CI 0.41-0.64; p<0.001)
HR 0.55 (95% CI 0.42-0.71; p<0.001)
Overall survival (%)
Overall survival (%)
p<0.001
p<0.001
Time (months)
Time (months)
| LD combined RT | 161 | 59 | 20 | 13 | 6 | 5 | 3 |
| --- | --- | --- | --- | --- | --- | --- | --- |
| Sorafenib | 186 | 36 | 13 | 5 | 3 | 1 | 1 |
| LD combined RT | 144 | 90 | 44 | 27 | 22 | 12 | 10 |
| --- | --- | --- | --- | --- | --- | --- | --- |
| Sorafenib | 119 | 40 | 22 | 16 | 11 | 9 | 7 |

## Slide 5
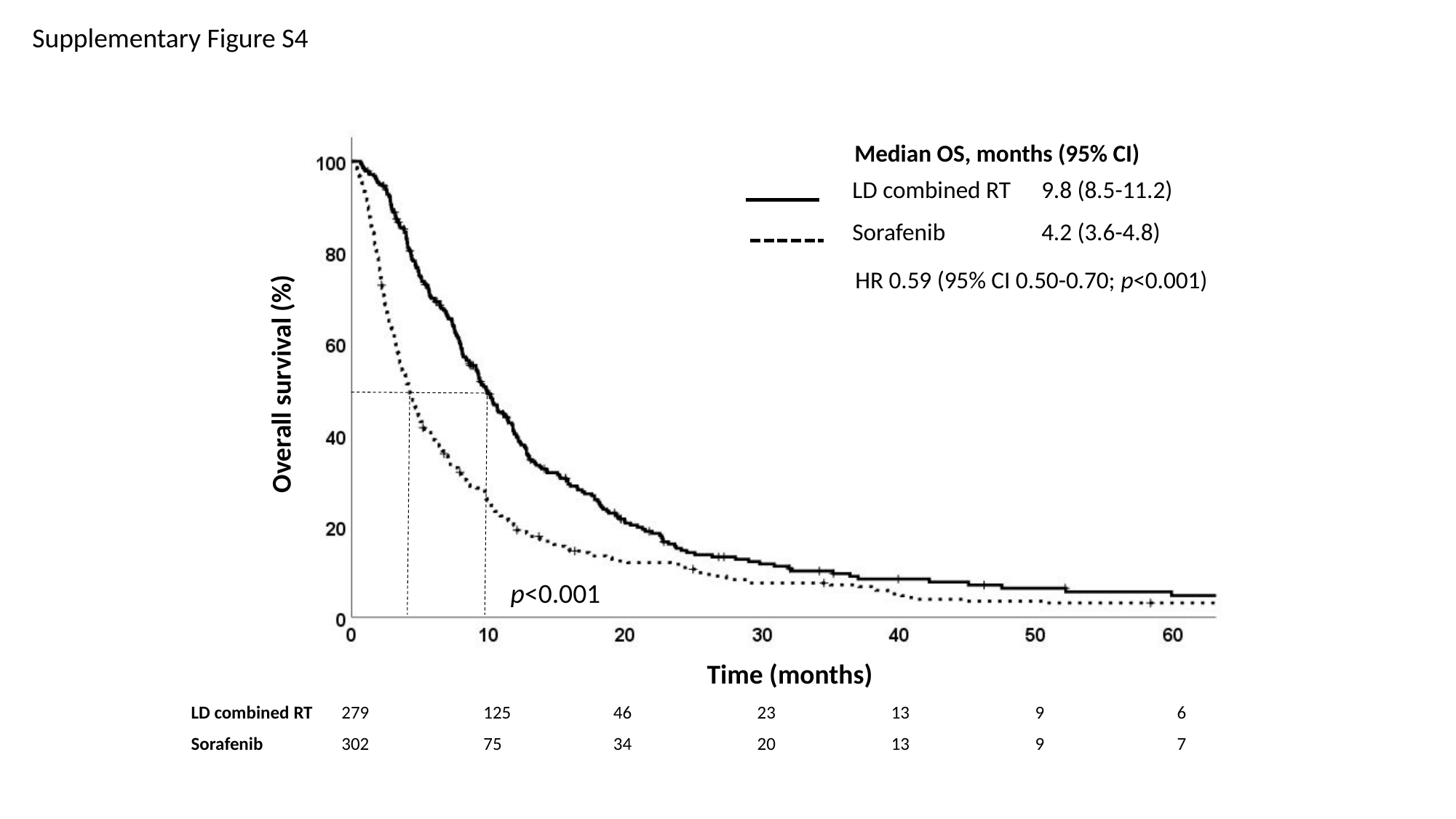

Supplementary Figure S4
Median OS, months (95% CI)
| LD combined RT | 9.8 (8.5-11.2) |
| --- | --- |
| Sorafenib | 4.2 (3.6-4.8) |
HR 0.59 (95% CI 0.50-0.70; p<0.001)
Overall survival (%)
p<0.001
Time (months)
| LD combined RT | 279 | 125 | 46 | 23 | 13 | 9 | 6 |
| --- | --- | --- | --- | --- | --- | --- | --- |
| Sorafenib | 302 | 75 | 34 | 20 | 13 | 9 | 7 |
