# Supplementary material for: Efficacy of Liver-Directed Combined Radiotherapy in Locally Advanced Hepatocellular Carcinoma with Portal Vein Tumor Thrombosis
Source: Cancers (Basel). 2023 Jun 13;15(12):3164. doi: 10.3390/cancers15123164 (PMC10296128; doi:10.3390/cancers15123164)
Supplement: Supplementary file 1 [file cancers-15-03164-s001.zip › cancers-2452888-supplementary/Supplementary Files/Supplementary Table.pdf]

Supplementary Table S1. Prognostic factors for progression-free survival before and after PSM

	Univariable analysis			Multivariable analysis		
	HR	95% CI	*p* value	HR	95% CI	*p* value
Before PSM						
Treatment (LD combined RT vs Sorafenib)	0.47	0.42-0.54	<0.001	0.40	0.35-0.47	<0.001
Sex (Female vs Male)	1.15	0.96-1.39	0.137	N.S.		
Age	1.00	1.00-1.01	0.532	N.S.		
ECOG PS (2-3 vs 0-1)	1.20	0.96-1.51	0.110	N.S.		
Child-Pugh class (B-C vs A)	1.88	1.63-2.18	<0.001	1.72	1.48-2.01	<0.001
Prior treatment history (Yes vs No)	1.15	1.00-1.32	0.052	N.S.		
Log(Pretreatment AFP)	1.20	1.14-1.25	<0.001	1.18	1.12-1.23	<0.001
Tumor size	1.03	1.02-1.04	<0.001	1.03	1.02-1.05	<0.001
Disease extent (Bilateral vs Unilateral)	1.49	1.31-1.69	<0.001	1.16	1.02-1.33	0.030
LN status (Involved vs Not involved)	1.34	1.12-1.62	0.002	1.22	1.01-1.48	0.039
PVTT type (III, IV vs I, II)	1.17	1.03-1.33	0.018	N.S.		
After PSM						
Treatment (LD combined RT vs Sorafenib)	0.47	0.40-0.56	<0.001	0.41	0.35-0.49	<0.001
Sex (Female vs Male)	0.96	0.75-1.23	0.736	N.S.		
Age	1.00	0.99-1.00	0.302	N.S.		
ECOG PS (2-3 vs 0-1)	1.42	1.06-1.91	0.019	N.S.		
Child-Pugh class (B vs A)	1.70	1.40-2.06	<0.001	1.55	1.26-1.90	<0.001
Prior treatment history (Yes vs No)	0.94	0.80-1.12	0.493	N.S.		
Log(Pretreatment AFP)	1.25	1.17-1.32	<0.001	1.22	1.15-1.30	<0.001
Tumor size	1.04	1.03-1.06	<0.001	1.04	1.02-1.06	<0.001
Disease extent (Bilateral vs Unilateral)	1.34	1.13-1.58	0.001	N.S.		
LN status (Involved vs Not involved)	1.40	1.09-1.80	0.010	N.S.		
PVTT type (III, IV vs I, II)	1.17	0.99-1.38	0.068	N.S.		

*Abbreviations: PSM, propensity score matching; HR, hazard ratio; CI, confidence interval; LD combined RT, liver-directed combined radiotherapy; ECOG PS, Eastern Cooperative Oncology Group performance status; AFP, alpha-fetoprotein; LN, lymph node; PVTT, portal vein tumor thrombosis; N.S., not significant
